# Curious Cerebral or Nervous Affection, of a Periodical Character

**Published:** 1829-01-01

**Authors:** 


### XXII.

### CURIOUS CEREBRAL, OR NERVOUS AFFECTION OF A PERIODICAL CHARACTER. By Dr. STRAMBIO.

The following case is worthy of attention, especially if taken in connexion with the Essay on Malaria, Tic Douloureux, &c. lately published by Dr. Macculloch, n this country.

*Case.* A man who was given to study, and who was subject to otitis, and ophthalmia, became exposed to a current of cold air, while asleep, and while making a tour on the shores of the lake of Como. From this moment, an ophthalmic discharge which he had, became suppressed, and was succeeded by severe pains (like tic douloureux) in the left side of the face. These pains came on for two or more hours each day, and then ceased. By these paroxysms he was harrassed occasionally during the Spring, Summer, and Autumn following. In the Winter he had long intervals from these attacks; but when they came on, they lasted from 24 to 62 hours at a time. They were, also, accompanied by rheumatic affections, which were treated by bleeding and regimen. The patient supported his complaints without much difficulty, till the month of October, 1824, when he was seized with violent convulsions. In proportion as these increased, the original pains diminished, and only returned three or four times per annum. The convulsions came on generally at particular times of the day, and after lasting for two or three hours, would cease. In June, 1827, they became almost uninterrupted, accompanied by the following phenomena: viz. unsteady gait—inability to look directly upwards or downwards, without risk of falling—alternate sensations of weight and emptiness in his head—wandering pains in the integuments of the face—a feeling of suffocation on any sudden change of temperature—wandering pains and sense of soreness in different parts of the body—pulsations in the chest and at the epigastrium—acute pains in the head, with violent ringing and beating there—constipation of the bowels—pale urine with white sediment—itching of the eyes—stinging and shootings in the soles of the feet and palms of the hands—startings and sense of anxiety on first going to sleep, after which, the sleep was sound—vertigo, with various spectral illusions. All these symptoms diminished considerably in the course of an hour after eating, and also after alvine evacuations. The patient had a morbid inclination for food, but the thirst was moderate.

He made a journey to the Waters of Recoaro; but, on his return, he suffered worse than ever, especially from a sense of constant drunkenness, increased by the least mental excitement, or corporeal movement. Attributing this feeling to debility, he augmented the quantity of his wine, but without benefit. On the contrary, he had pains along the spine and in his shoulders—his gait was vacillating—he broke out into perspirations on the least exertion.

Dr. Strambio being consulted, attributed the complaint to a congestion, probably with serous effusion in and between the membranes of the brain and spinal marrow. With this view, he ordered local and general bleeding—a rigid system of diet—abstinence from wine and other stimulants—and a caustic issue in the neighbourhood of the cervical vertebræ. By perseverance in these means for two months, the patient so far recovered as to be able to resume his usual avocations, and in March, 1828, he was pronounced to be cured.

Notwithstanding the success which followed Dr. Strambio's depletive treatment, in this case, we imagine that most pathologists in this country will be inclined to view the disease as one of the nervous system, rather than an inflammatory affection of the cerebral and spinal membranes. We are sure that Dr. Macculloch would attribute the complaint to malaria applied to the body, when asleep on the shores of the Como. The periodical character which it assumed for so long a time, inclines us to think, that Dr. M.'s doctrine would apply in this case. But our readers will form their own conclusions.—*Giorn. Analeteo de Medicina.*

---

